# Impedimetric Characterization of NanA Structural Domains Activity on Sialoside-Containing Interfaces

**DOI:** 10.1021/acs.langmuir.4c02620

**Published:** 2024-10-08

**Authors:** Israel Alshanski, Suraj Toraskar, Karin Mor, Franck Daligault, Prashant Jain, Cyrille Grandjean, Raghavendra Kikkeri, Mattan Hurevich, Shlomo Yitzchaik

**Affiliations:** † The Institute of Chemistry and Center of Nanotechnology, The Hebrew University of Jerusalem, Jerusalem 91904, Israel; ‡ Indian Institute of Science Education and Research, Dr. Homi Bhabha Road, Pune411008, India; § Nantes Université, CNRS, US2B, UMR 6286, F-44000 Nantes, France; ∥ Singapore-HUJ Alliance for Research and Enterprise (SHARE), The Cellular Agriculture (CellAg) Programme, Campus for Research Excellence and Technological Enterprise (CREATE), 138602 Singapore

## Abstract

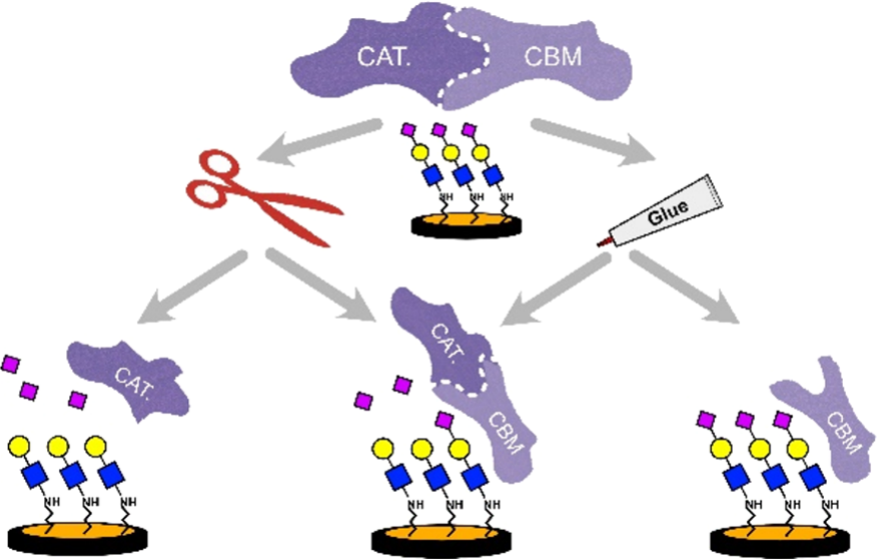

*Streptococcus pneumoniae* is a pathogenic bacterium that contains the surface-bound neuraminidase, *NanA*. *NanA* has two domains that interact with sialosides. It is hard to determine the contribution of each domain separately on catalysis or binding. In this work, we used biochemical methods to obtain the separated domains, applied electrochemical and surface analysis approaches, and determined the catalytic and binding preferences toward a surface-bound library of sialosides. Impedimetric studies on two different surfaces revealed that protein–surface interactions provide a tool for distinguishing the unique contribution of each domain at the interface affecting the substrate preference of the enzyme in different surroundings. We showed that each domain has a sialoside-specific affinity. Furthermore, while the interaction of the sialoside-covered surface with the carbohydrate-binding domain results in an increase in impedance and binding, the catalytic domain adheres to the surface at high concentrations but retains its catalytic activity at low concentrations.

## Introduction

Electrochemical impedance spectroscopy (EIS) is a label-free analytical technique that can be used to sense changes caused in a solid–liquid interface such as interactions with ions, molecules, proteins, cells, enzymatic reactions, and organic reactions.^1−6^ The changes in the layer properties such as density, morphology, and interfacial charge are translated by EIS to changes in capacitance and charge transfer resistance (*R*_CT_).^6−8^ EIS can be used to study enzyme–substrate interactions.^9^ When the substrate is anchored to the electrode, the enzyme is the analyte. In such electrochemical systems, the substrate-bound electrode can probe either the binding or the catalysis of the enzyme from the solution.^4,8^

Glycans make up a family of molecules decorating the extracellular matrix. They serve in many communication and signaling pathways. Sialic acid (SA) is a unique monosaccharide containing nine carbons in the backbone.^10,11^ SA is usually attached to galactose or galactosamine to form a family of glycans known as sialosides.^12^ SA expression can be found at the cell surface and is essential for cell communication and interactions.^10,11^ Sialosides are prime targets for pathogen cell invasion. Pathogens have unique proteins that can either bind or catalyze SA enzymatic reactions on cell surface.^13^ Therefore, it is important to monitor SA-related enzymatic processes on glycans.

Studying glycan interactions using conventional biochemical methods stems from inaccessibility to substantial amounts of glycans’ low binding affinity and the lack of significant heat evolution or spectroscopic changes associated with the binding. Glycan monolayers on electrodes can serve to study many types of interactions spanning from small ions to proteins.^3,8,14−19^ Impedimetric methods have been perfected for studying glycan interactions. Since they rely on changes in the dielectric properties of the glycan-monolayer, no labeling is required, there is no need to follow heat or optical changes, and only a small amount of the glycan is needed. Recently, we have demonstrated that electrodes containing sialylated glycans monolayers allow for studying enzymatic processes that involve SA.^4^ We used EIS to show that both enzymatic sialylation (using sialyltransferase) and SA hydrolysis (using neuraminidase and NA) can be followed. Pathogens’ interactions with SA are a key step in their infectivity, resistivity, and mode of action. Desialylation reactions in viruses and bacteria take place by using pathogen-specific NAs. For enzymes that contained both catalytic and binding domains, the EIS studies resulted in a complex mode of interaction that was influenced by both the sialoside substrate and the electrode surface. The interplay between the surface characteristics and the sialoside structure is crucial for sensing application. There are various ways by which sensing applications can benefit from these combined features. The sialoside structure provides a NA preference that can lead to specificity based on the native architectures of the carbohydrate-binding domain (CBD) or of the catalytic pocket. The surface of the electrode and the submonolayer surrounding the sialoside can also interact with the protein. The interaction of the region that surrounds the carbohydrate interacting domains in the parent protein with the surface around the glycan is also specific and can result in either additional efficacy or repulsion. This results in a surface-controlled mode of interaction, which can provide sensing either through catalysis or via a binding mechanism. This enables controlling the NA interaction mode and specificity in EIS studies of sialosides bound to variable electrode surfaces.^8,14^

While in many pathogens the sialoside binding protein differs from proteins involved in catalysis, in other organisms, the same protein contains both functions. Many NAs have both binding and catalytic domains. In some cases, the catalytic site can also be involved in binding.^8,14^ For NAs that contain both functions, it is crucial to determine the contribution of each domain to the observed electrochemical response. In our previous efforts, we established a strategy to differentiate between these functions by utilizing different electrodes with variable surface chemistries.^8,14^ Until now, wild-type (WT) NAs containing both domains were evaluated using our EIS strategy; hence, their individual contribution to the interaction mode remains elusive. To study the effect of each domain separately, a truncated protein should be produced with high fidelity, containing only one specific function.

**Figure 1:**
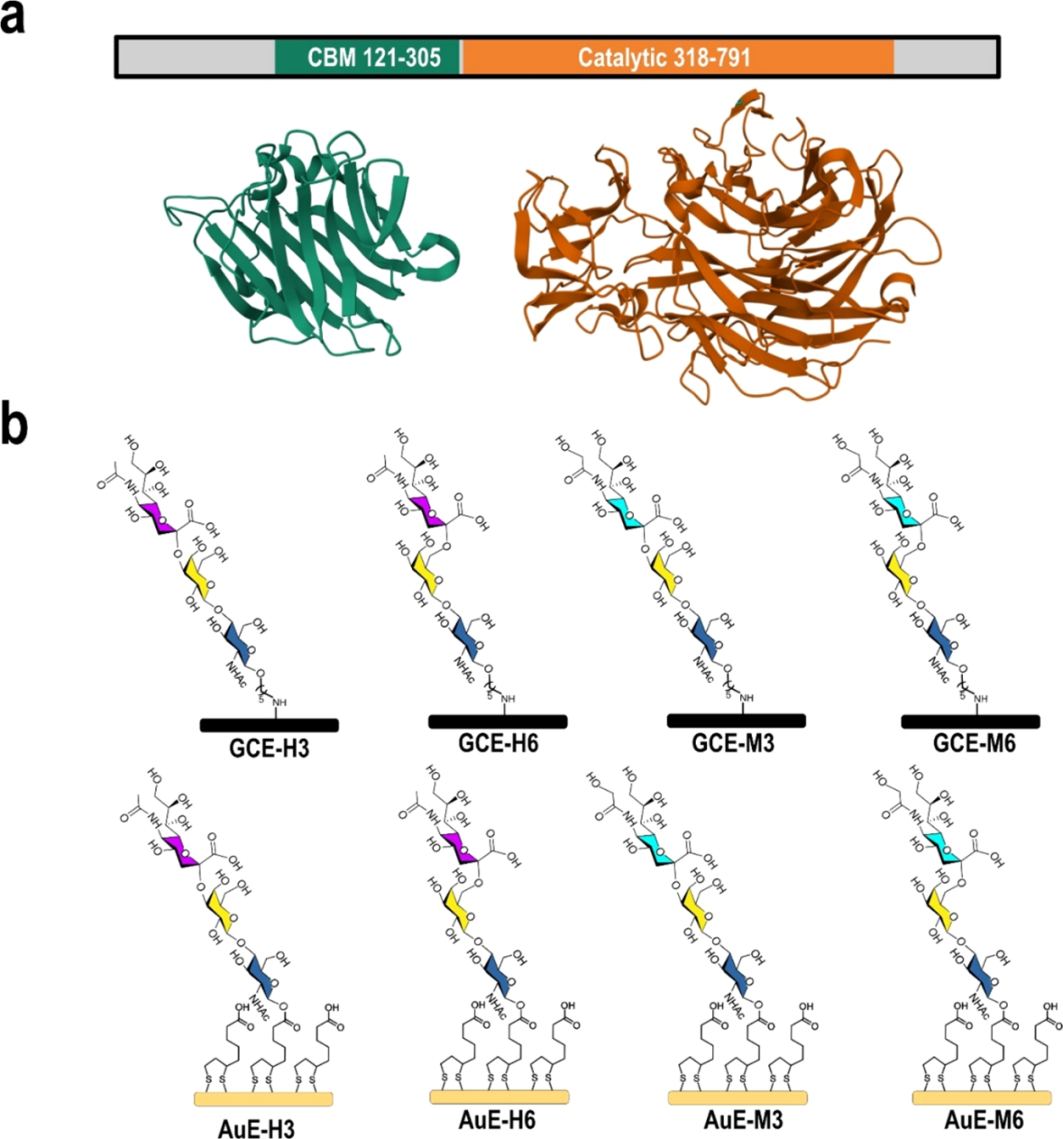
(a) Schematic domain of *NanA* with a focus on the CBM and the Cat domains with the structure of CBM (green, PDBiD 4ZXK)^27^ and Cat (Brown, PDBiD 2VVZ).^29^ (b) Sialoside-modified electrodes used in this study. The submonolayer of the GCE (black) comprises hydrophobic interface around the sialoside which contributes to the binding interactions with NAs. The LPA submonolayer surrounding the sialoside on AuE (yellow) introduces electrostatic repulsions, which benefits catalysis over binding in NAs.

*Streptococcus pneumoniae* is a Gram-positive bacterial pathogen that is a major cause of respiratory infection, otitis media, pneumonia, meningitis, and septicemia.^20−22^*S. pneumoniae* expresses NA membrane-bound (*NanA*),^23^ which plays a major role in infection.^24^*NanA* substrates include sialosides containing α2,3 and α2,6 *N*-acetyl neuraminic acid (Neu5Ac). The crystallographic structure of *NanA* shows that the enzyme contains two domains that interact with SA. The first domain is the carbohydrate-binding module (CBM, residues 121–305) which binds SA-terminated glycans, the second one is catalytic (Cat, residues 318–791) responsible for hydrolyzing the SA (Figure 1a).^25−28^ Although the structure and role of each domain of the enzyme are known, the contribution of the CBM domain to the catalytic activity of the enzyme is unknown.

We hypothesized that by taking advantage of our established NA EIS analysis strategy and accessibility to truncated *NanA* fragments, the interplay between activity and affinity of each domain can be elucidated.^8^ Herein, we used sialosides attached to either gold or glassy carbon electrodes (GCE) to profile the sialoside preference of WT *NanA* and elucidate the involvement of each separated domain in those interactions.

## Results and Discussion

In previous works, we showed that the binding preference of neuraminidase can be evaluated on GCE modified with sialosides while enzymatic activity can be evaluated on AuE modified with sialosides.^8^ To evaluate the preferences of NanA for the sialoside type at an interface, GCE and AuE were modified with four sialoside trisaccharides. The trisaccharides contain the same disaccharide core structure of galactose-glucosamine and differ in the SA type and connectivity. We systematically named the trisaccharides. Sialosides containing *N*-acetyl neuraminic acid were abbreviated with H, as they are associated with human glycomics. Sialosides containing *N*-glycolylneuraminic acid were abbreviated with M, as they are associated with nonhuman mammalian glycomics. The number following the letter refers to the connectivity of the SA to the galactose, where 3 defines 2,3 connectivity and 6 stands for 2,6 connectivity. The following types were hence abbreviated as follows: 2,3 Neu5Ac (**H3**), 2,6 Neu5Ac (**H6**), 2,3 *N*-glycolylneuraminic acid (Neu5Gc) (**M3**), and 2,6 Neu5Gc (**M6**) (Figure 1B). This enabled determining the unique response mode, binding or catalysis, of different NAs toward the glycan-bound electrode set.^14^

**Figure 2:**
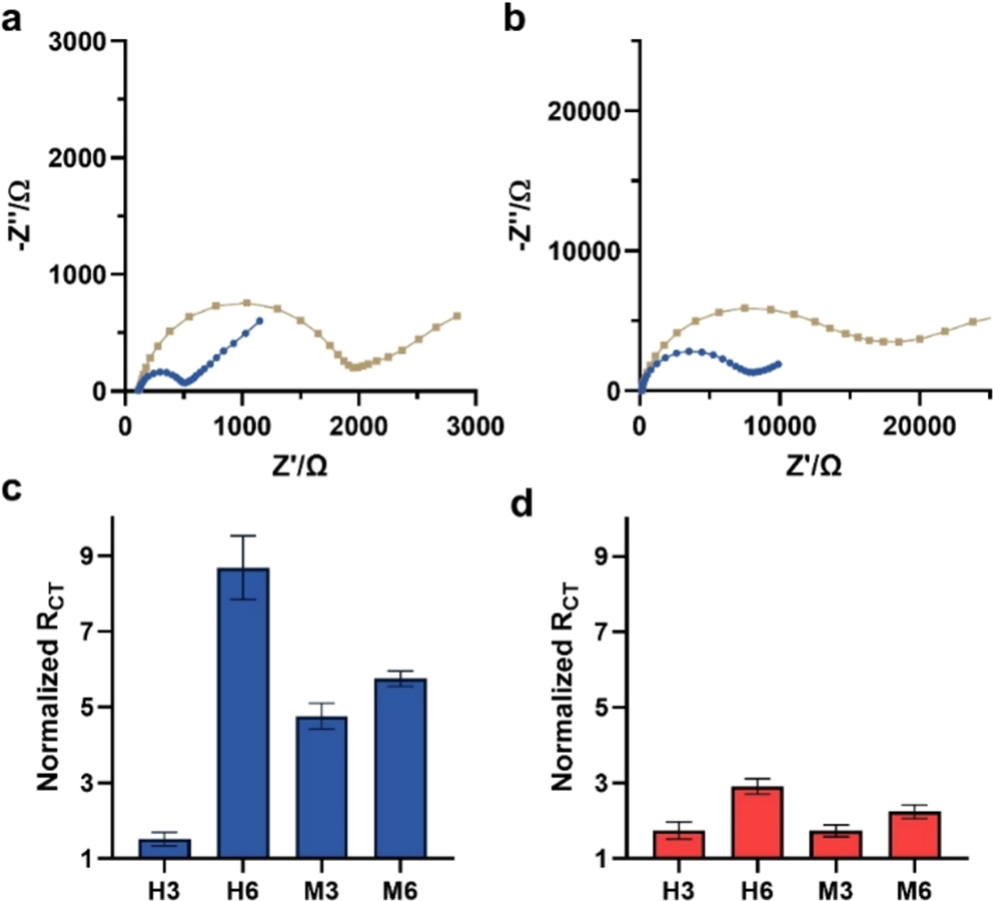
EIS response of WT *NanA* to the eight different sialoside-anchored electrodes. Nyquist plot of **GCE-H6** (a) and **AuE-H6** (b) prior (blue) and after (brown) exposure to WT *NanA*. Normalized *R*_CT_ for the response of GCE-sialosides (c) and AuE-sialosides (d) toward WT *NanA*. Error bars are based on five electrodes.

To evaluate the response mode of WT *NanA* (Residues 121–791), the enzyme was incubated with eight different sialoside-anchored electrodes and the charge transfer resistance (*R*_CT_) change was recorded (Figure 2). EIS analysis showed that sialoside has a higher density on the gold electrode compared to the glassy carbon one (e.g., H6 Figure 2a,b). For both surfaces, incubation with WT *NanA* resulted in an *R*_CT_ increase. The change in *R*_CT_ on the GCE was larger than that on AuE. There is a clear sialoside-derived preference. The response to **H6** was the highest on both surfaces compared to the other sialosides (Figure 2c,d). Our results show that the affinity toward the 2,6 linked sialosides is larger than for the 2,3 sialosides for both Neu5Ac and Neu5Gc. *NanA* showed high binding affinity to both **AuE-H6** and **GCE-H6**, which indicates that the affinity of WT *NanA* is high to 2,6 Neu5Ac substrate in the two interfaces. Additionally, this NA has almost negligible response toward the 2,3 Neu5Ac substrate, which is surprising because enzyme analyses in solution showed no preferences for NanA activity.^27,28,30^ This indicates that the interface has a crucial effect on the enzyme sensing selectivity and can highlight the preferences of the enzyme and binding/activity mode of action on the surface. It is important to note that the enzyme response is dissimilar to the other bacterial and viral NA, which were analyzed in our previous work.^14^

**Figure 3:**
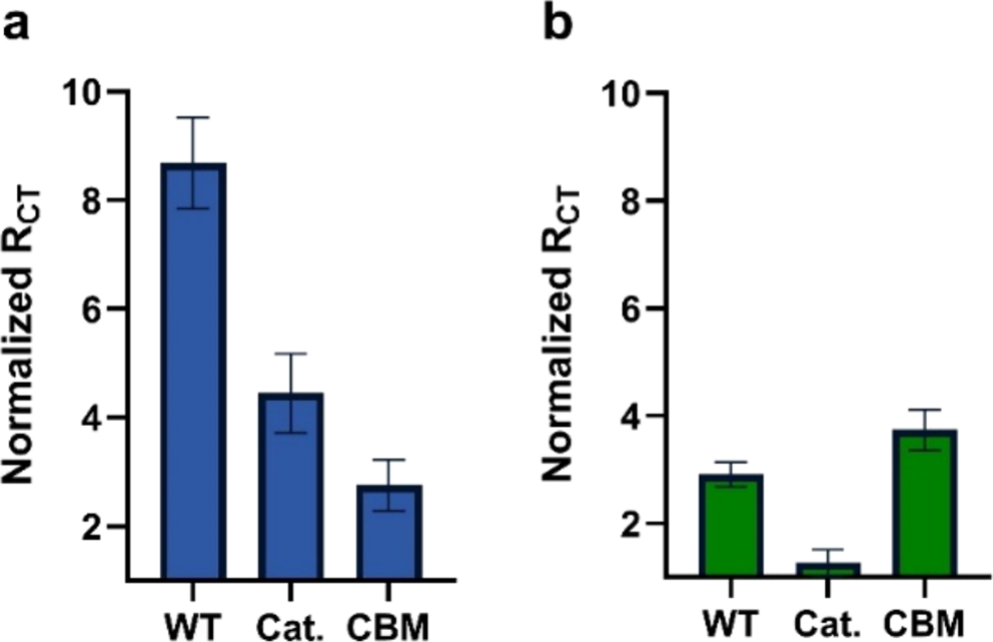
Normalized *R*_CT_ for the response of GCE-H6 (a) and AuE-H6 (b) to the exposure of the electrode to the WT, Cat Domain, and CBM of NanA. Error bars are based on five electrodes.

In previous studies, we proved that NA binding to sialoside-anchored surfaces results in an *R*_CT_ increase, while hydrolysis from such surfaces leads to an *R*_CT_ decrease.^8^ To evaluate the contribution of each *NanA* domain to interactions with sialosides, **GCE-H6** and **Au–H6** were incubated separately with the CBM and the Cat domains, and the impedimetric response was compared to that of the WT (Figure 3). When each domain was exposed to **GCE-H6**, an increase in impedance was observed. The change in *R*_CT_ recorded for the WT was larger than that for the two separate domains. The sum of the impedimetric response to Cat and CBM was smaller than that of the WT protein. We suggest that the stronger response originates from the higher *M*_W_ of the full protein and the presence of the two recognition sites. For the two truncated domains with only one recognition site, the Cat domain has a stronger response than the CBM domain. This can be attributed to structural differences between the domains. The Cat domain is larger than the CBM (Figure 1A and references therein). The larger footprint of the Cat domain induces a stronger impedimetric response. The SA recognition site of the Cat segment is deeper than the one of the CBM binding pocket which can result in stronger binding to the sialoside-anchored GCE surface.

Exposure of the different domains to **AuE-H6** results in different behavior than on GCE. The increase of *R*_CT_ resulting from incubation with the CBM domain was significantly higher than toward the Cat domain and slightly higher than the WT (Figure 3b). In previous work, we observed a decrease in *R*_CT_ following incubation with NA. This was attributed to the enzymatic hydrolysis of the SA on the sialoside-anchored LPA-AuE monolayer.^8^ Here, the Cat domain does not show the expected decrease in *R*_CT_, which is usually associated with hydrolysis, while the CBM shows an increase in *R*_CT_, which is usually associated with binding. The increase in *R*_CT_ observed for both the WT and CBM indicates that the interaction of both is governed predominantly by binding. The slightly lower impedimetric response of the WT can be attributed to a partial catalytic activity. Putting together these results with previous observation suggest that the increase in impedance resulted from protein binding and that the WT has two competing activities on the surface that result in a smaller response compared to the CBM domain which can only bind sialic acid. To clarify if the observed impedimetric response recorded on gold surfaces correlates with the amount of protein on the surface, X-ray photoelectron spectroscopy (XPS) was performed. XPS analyses of the N_1S_ (SI) signal at 400.1 eV show there is a large increase of the amide region for all domains, indicating that the enzyme was adsorbed to the surface. Quantification of the amide’s amounts indicated that the amount of CBM domain on the surface is 7 times greater than that of WT and the difference is even higher compared to the Cat domain. This explains the variation in *R*_CT_ increase in response to each domain of the enzyme.

**Figure 4:**
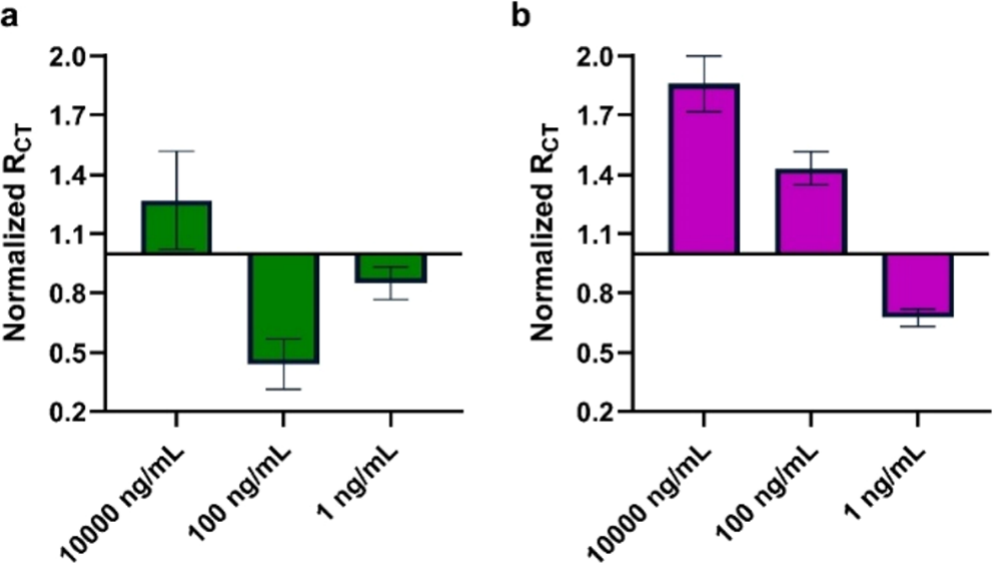
Normalized *R*_CT_ of the effect of Cat domain concentration on the activity of **AuE-H6** (a) and **AuE-H3** (b).

Sialoside-modified AuE electrodes showed a decrease in *R*_CT_ resulting from enzymatic activity.^8,14^ Since the impedimetric measurement of the Cat domain did not show the same catalytic trend as other reported NA, we examined the concentration-dependent effect of the Cat domain on the impedimetric response (Figure 4a). The expected decrease in *R*_CT_ is observed only in low Cat concentrations (1–100 ng/mL), while at a very high concentration (1 μg/mL), the impedance increases. At low concentrations, the behavior is normal for enzymatic catalysis and decreases with lowering the enzyme concentration. The results show that at low concentrations, the activity is slow and results in a small change in *R*_CT_. At high concentrations, there is a competition between the enzymatic activity and the more dominant interface associated binding that leads to an increase in *R*_CT_. In a concentration between the two boundaries, there is an observable enzymatic activity that was also confirmed by the N 1s signal in XPS (SI).

The above results suggest that the *NanA* enzyme has activity at the interface of the AuE. Additionally, **AuE-H3** was exposed to various concentrations of the Cat domain to compare the preference of the domain (Figure 4b). The Cat domain showed a stronger affinity to **AuE-H3** and activity at lower concentrations compared to **AuE-H6**. This contrasts with that of the WT NanA, which has a higher affinity to **AuE-H6**. At high concentrations, the Cat domain shows an increase in impedance for both sialosides on gold. This suggests that the binding masks the catalytic activity. We cannot exclude that there is a constant equilibrium between binding and catalysis. Such a phenomenon can be observed at two extremes; when the concentration is extremely high, the Cat remains attached to the surface thereby increasing the impedance. At low concentrations, catalysis releases enough protein from the surface to cause a significant decrease in the *R*_CT_. It might be that the binding/catalysis equilibrium is sialoside-type-dependent and those differences manifest themselves in different preferences on the surface in this study. The different preferences can be a result of the combined interaction between the enzyme-electrode interface in addition to that with the substrate. For instance, the interface can induce the preference for sialic acid by having stronger or weaker interactions with the enzyme upon recognition event and in some cases even prevent enzyme detachment as in the case of GCE. These results emphasize the importance of each in the activity of the full enzyme and the importance of the environment in which the enzymatic activity is carried out.

**Figure 5:**
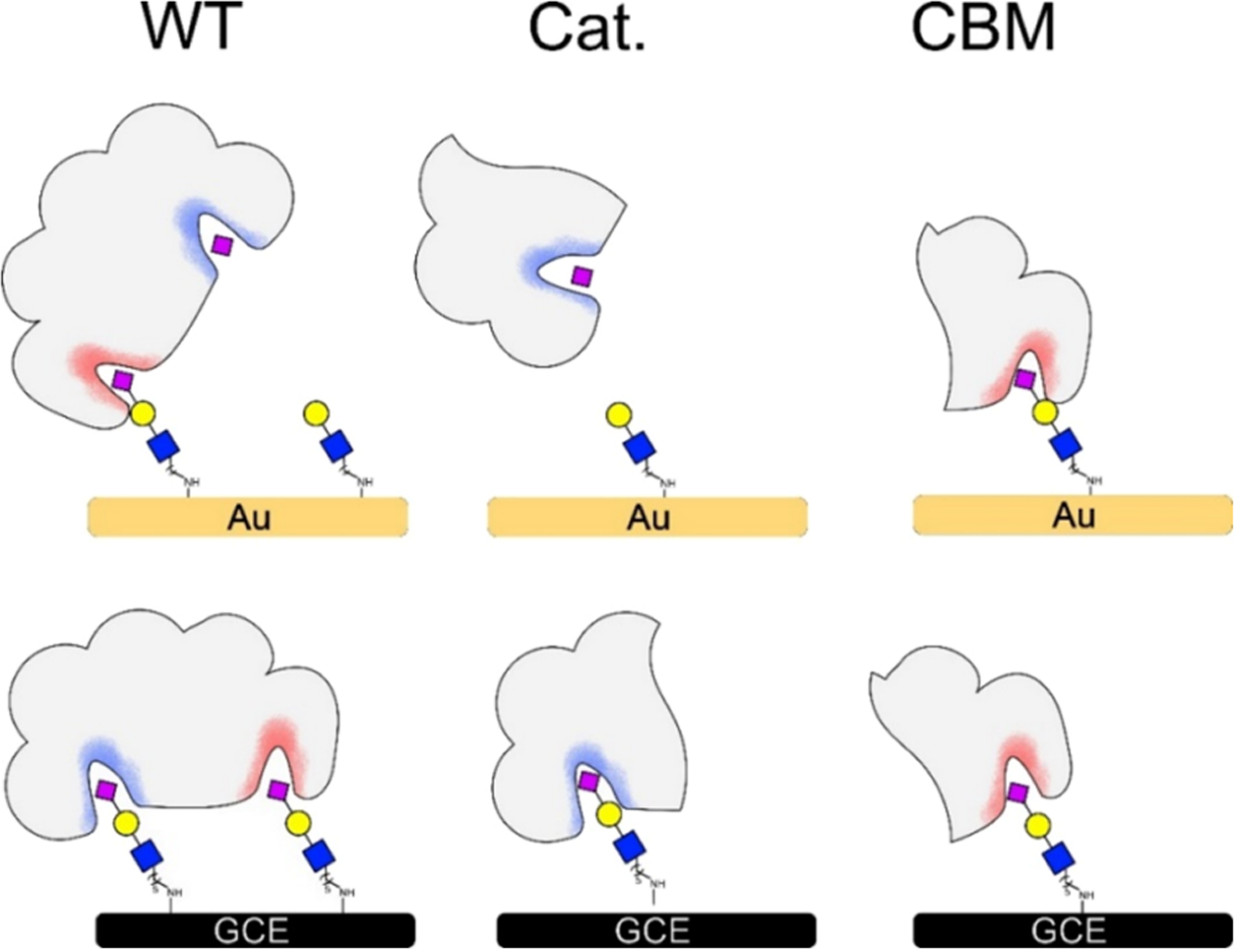
Schematic representation of the interaction of *NanA* domains with sialosides on the two interfaces. The WT (left) contains both the catalytic domain (blue) and the CBD (red). The recombinant fragments contain either the catalytic domain (middle) or the CBM (right). On AuE-modified interface (top), the Cat domain performs an enzymatic reaction, the CBM binds the sialoside and the WT has contribution of both domains. On GCE (bottom), all of the NanA constructs experience interactions with the submonolayer that leads to binding regardless of the domain’s nature.

The collective results from the system suggest the different behaviors of the WT *NanA* and the domains at the various interfaces of the electrodes (Figure 5). In the case of the GCE interface, each domain interacts with the sialoside, which causes binding to the surface. The WT enzyme has the contribution of both domains for binding, which increases the binding affinity. On the other hand, in AuE, the Cat domain has activity while the CBM domain has binding affinity. In this case, the WT domain has competing activities of the two domains, which result in signal change between the two domains separately. It is also important to note from the results that the presence or absence of part of the enzyme can affect the function of the enzyme. The resulting observations demonstrate that impedimetric tools can help in determining the contribution of each domain to the activity of an enzyme.

Many methods were developed to study sialoside hydrolysis. Kinetic studies in solution usually rely on labeled sialosides and overlook the fact that in nature those entities are bound to proteins, lipids, or an extended glycan chain. The environment around the sialosides introduces constraints that might contribute to a kinetic preference. Our study shows that there is a combined effect of the seaside-type and surface features. We utilized these designed constraints and observed 2,3/2,6 sialoside-NanA preferences that were not recorded in solutions.

## Conclusions

*NanA* is a crucial surface-bound NA of pathogenic *S. pneumonia*, which has a large variety of substrates. *NanA* can be divided into two domains that interact differently with the substrates: CBM domain, which binds sialic acid, and Cat domain, which cleaves sialic acid. In this work, we showed the activity of each domain on the interface by EIS. CBM binds sialoside at the interfaces of GCE and AuE, while the Cat domain binds to modified GCE and performs catalysis at the interface of the modified AuE. We demonstrated the combined effect of the domains on the *NanA* response at the interface and the importance of enzyme-interface interactions in enzyme evaluation. In the case of the modified AuE interface, the two domains have competing effects, while in the case of the GCE-modified interface, the two domains have a synergistic effect. Additionally, we showed that *NanA* has a different response to the modified interface of sialoside than other bacterial and viral NA; hence, the platform can be used also for the detection of *NanA*. These observations show that the role of enzyme domains in activity can be evaluated using EIS on the differently modified interface and that the technique can be used for selective biosensing.

## Experimental Section

### Materials

All materials were purchased from Merck.

### Sialoside Synthesis

Sialoside trisaccharides were synthesized according to our previously reported procedure.^8^

### NanA Expression and Purification

NanA-WT protein (residues 121 to 791) corresponds to the original full-length NanA sequence without the signal peptide (residues 1 to 52), a disordered domain (residues 53 to 120), and the C-terminal sequence (residues 791 to 1035). The lectin (NanA-CBM) and the catalytic (NanA-Cat) domains comprise the residues from 121 to 305 and from 318 to 791 of the native NanA sequence, respectively.^31^ The gene encoding the three protein sequences was synthesized and optimized for *Escherichia coli* expression (GeneScript). They were next cloned into the pET19b plasmid into NdeI and XhoI sites in fusion with an N-terminal 10 Histidine tag. These plasmids were transformed into *E. coli* BL21(DE3) strain. Overnight 10 mL precultures in LB medium were diluted in 500 mL of LB medium supplemented with 1 μg/mL ampicillin and incubated at 37 °C until they reached an OD_600_ of 0.6. Then, the expression of the proteins was induced by the addition of IPTG (250 μL of a 1 M solution), and the cultures were incubated at 20 °C overnight. Bacterial pellets were lysed by sonication in lysis buffer (NaH_2_PO_4_ 50 mM, NaCl 150 mM, imidazole 5 mM, lysozyme 1 mg/mL, PMSF 1 mM, and DNase 1 μg/mL). The NanA-derived proteins were then purified by affinity chromatography from the clarified lysate by using NiNTA beads and an imidazole gradient. The proteins were analyzed for their purity by 12% sodium dodecyl-sulfate polyacrylamide gel electrophoresis (SDS-PAGE) electrophoresis, and protein concentration was determined using Bradford assay. Proteins were extensively dialyzed against H_2_O pH 7 (pH adjusted by the addition of ammonium hydrogen carbonate) and freeze-dried in the presence of trehalose (30% w/v) as a cryoprotectant.

### Preparation of Modified GCE and AuE and Electrochemical Measurements

Preparation of modified GCE and AuE and electrochemical measurements were performed by our previously reported procedure.^8^

### Exposure to the Enzyme

Stock samples of 1 mg of NA were dissolved in 1 mL of 50 mM acetate buffer (pH 5) to give a concentration of 1 mg/mL. 2 μL of each stock was added to 198 μL of 50 mM acetate buffer giving a final volume of 0.2 mL (10 μg/mL) for experiments with more diluted concentrations, and the dilution was performed by the same method. Each modified electrode was drop-cast with 50 μL of the solution for 1 h at 37 °C. After exposure, the electrodes were rinsed with the acetate buffer and measured by EIS.

### Surface Modification and XPS Analyses

Modification of surfaces with the sialosides and XPS analyses were performed by our previously reported procedure.^8^

## Supporting Information Available

EIS Nyquist plots and XPS data (PDF)
