# Supplementary material for: Impedimetric Characterization of NanA Structural Domains Activity on Sialoside-Containing Interfaces
Source: Langmuir. 2024 Oct 8;40(42):22152–8. doi: 10.1021/acs.langmuir.4c02620 (PMC11500401; doi:10.1021/acs.langmuir.4c02620)
Supplement: Supplementary file 1 — la4c02620_si_001.pdf [file la4c02620_si_001.pdf]

Supporting Information

Impedimetric characterization of NanA structural domains activity on sialoside-containing interfaces

Israel Alshanski,[a] Suraj Toraskar, [b] Karin Mor,[a] Franck Daligault,[c] Prashant Jain,[b] Cyrille Grandjean,[c] Raghavendra Kikkeri,[b*] Mattan Hurevich,[a*] Shlomo Yitzchaik[a*]

[a] The institute of chemistry and Center of Nanotechnology, The Hebrew University of Jerusalem. Jerusalem, 91904, Israel.

[b] Indian Institute of Science Education and Research, Dr. Homi Bhabha Road, Pune-411008, India.

[c] Nantes Universite, CNRS, US2B, UMR 6286, F-44000, Nantes, France.

Contents

XPS and electrochemical results 3
- XPS Analyses 3
- Nyquist Plot of GCE sialosides with NanA-WT: 4
- Nyquist Plot of AuE sialosides with NanA-WT: 5
- Nyquist Plot of GCE-H6 with Cat domain and CBM domain: 7
- Nyquist Plot of AuE-H6 with Cat domain and CBM domain: 8
- Nyquist Plot of AuE-H6 with Diluted Cat domain: 9
- Nyquist Plot of AuE-H3 with Cat domain: 10

XPS and electrochemical results

XPS Analyses

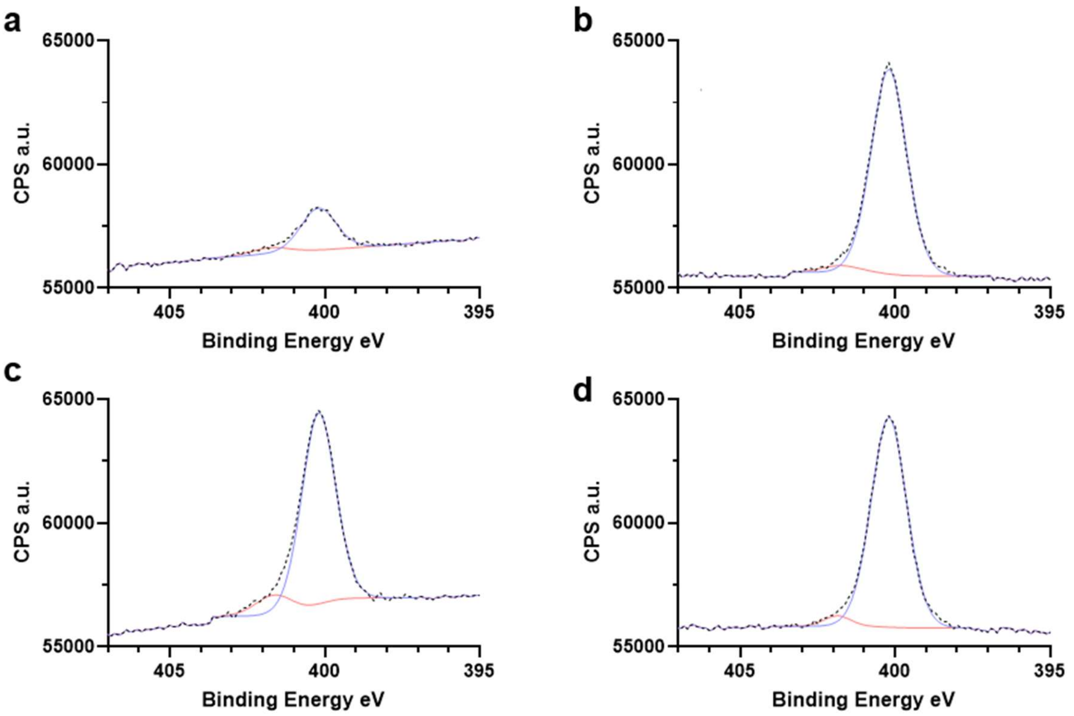

Figure S1: XPS analyses of N1S for Au-H6 prior (a) and after exposure to NanA-WT (b), Cat domain (c), and CBM domain (d).

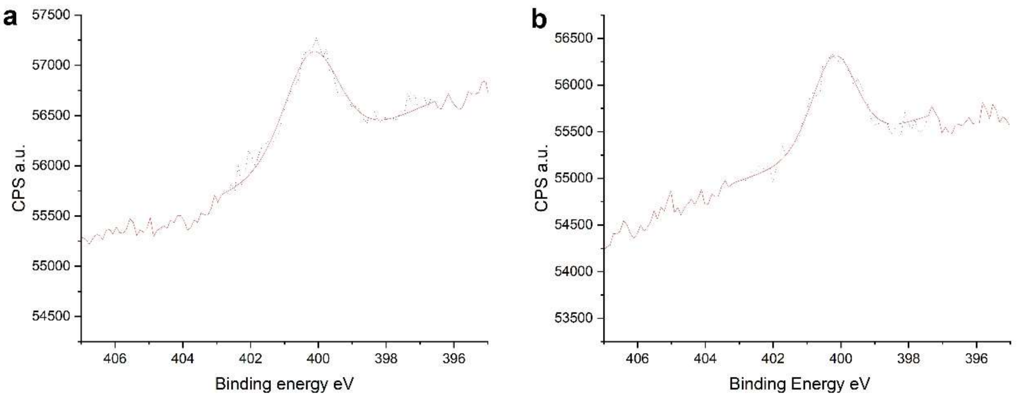

Figure S2: XPS analyses of N1S for Au-H6 prior (a) and after exposure to NanA-WT 100 ng/mL Cat domain (b).

Nyquist Plot of GCE sialosides with NanA-WT:

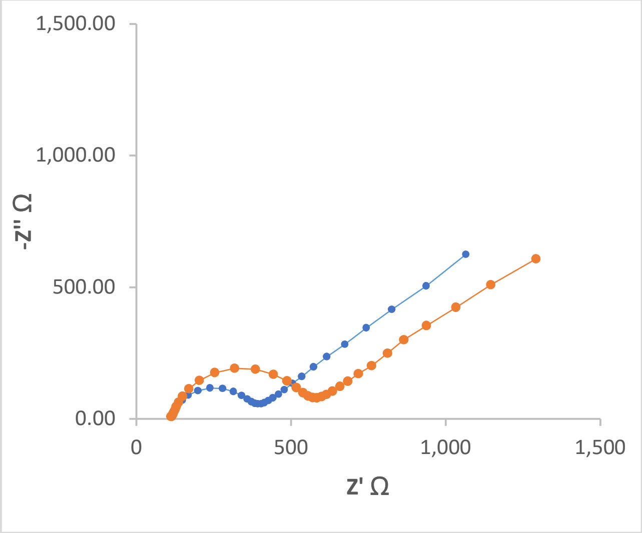

Figure S3: Nyquist plot of GCE-H3 prior (blue) and after (orange) exposure to WT-NanA.

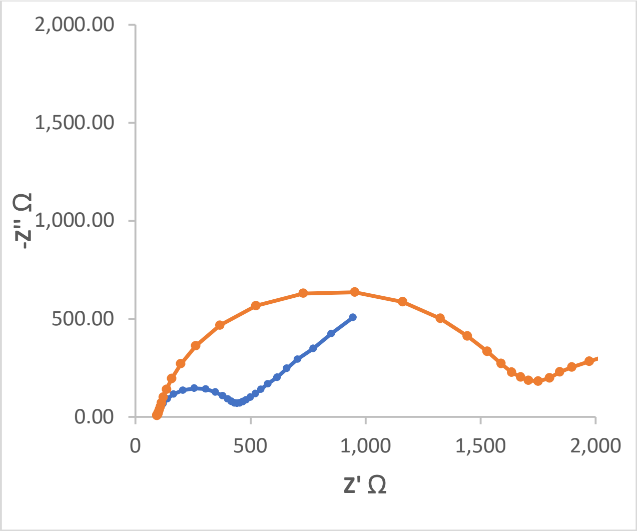

Figure S4: Nyquist plot of GCE-M3 prior (blue) and after (orange) exposure to WT-NanA.

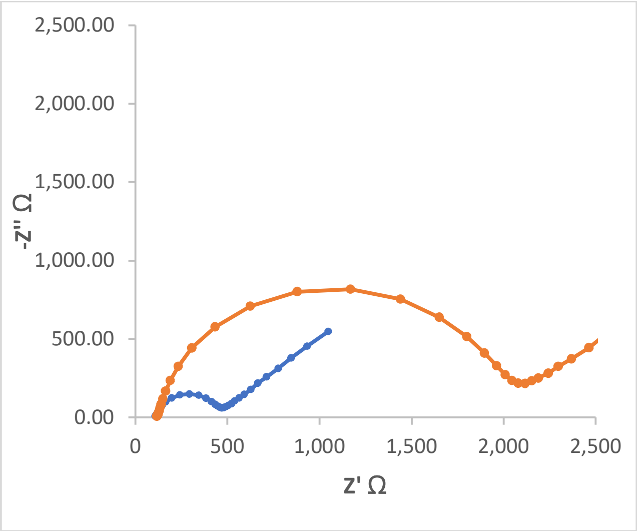

Figure S5: Nyquist plot of GCE-M6 prior (blue) and after (orange) exposure to WT-NanA.

Nyquist Plot of AuE sialosides with NanA-WT:

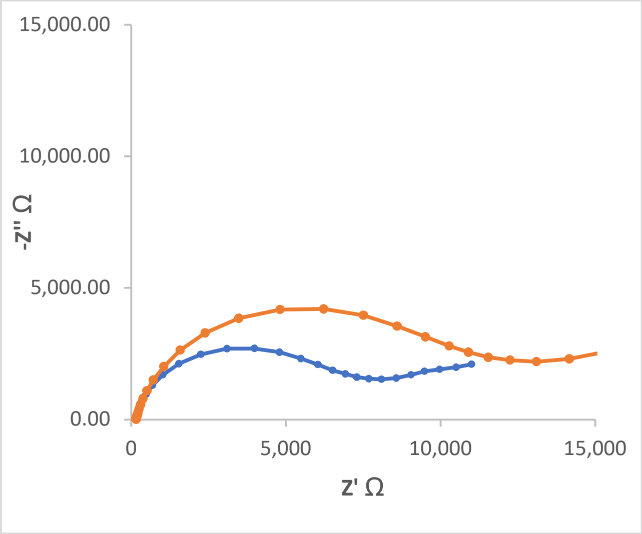

Figure S6: Nyquist plot of AuE-H3 prior (blue) and after (orange) exposure to WT-NanA.

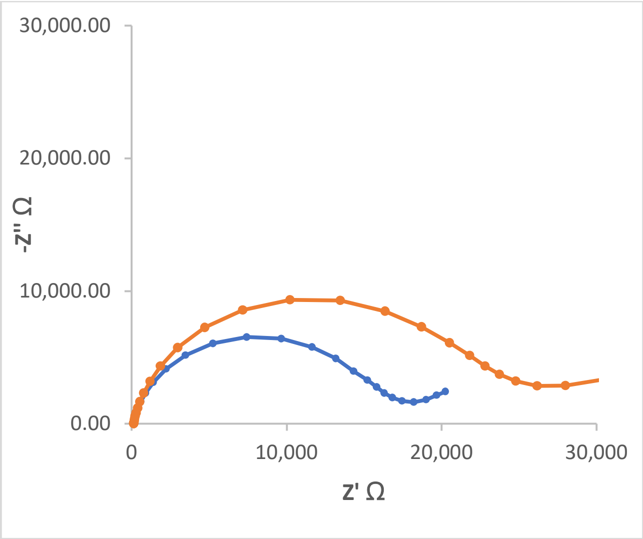

Figure S7: Nyquist plot of AuE-M3 prior (blue) and after (orange) exposure to WT-NanA.

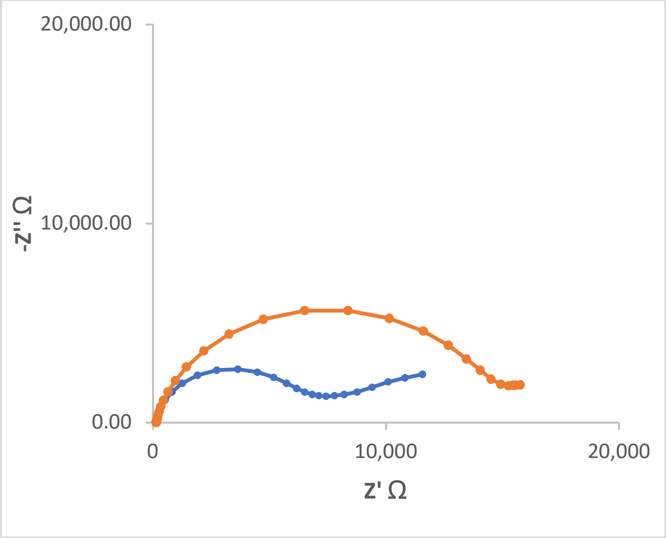

Figure S8: Nyquist plot of AuE-M6 prior (blue) and after (orange) exposure to WT-NanA.

Nyquist Plot of GCE-H6 with Cat domain and CBM domain:

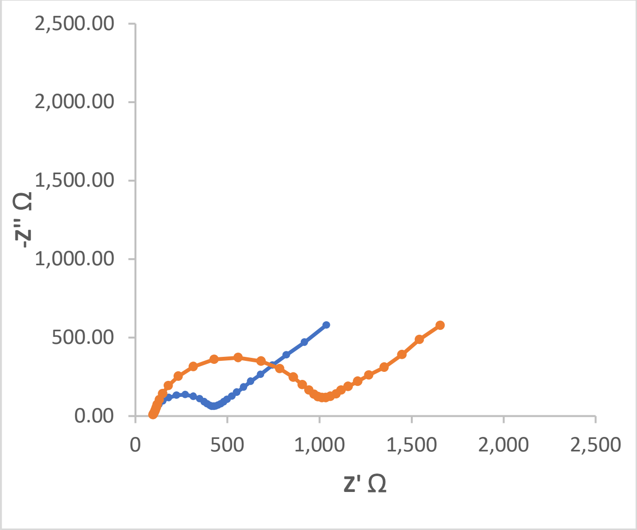

Figure S9: Nyquist plot of GCE-H6 prior (blue) and after (orange) exposure to CBM domain.

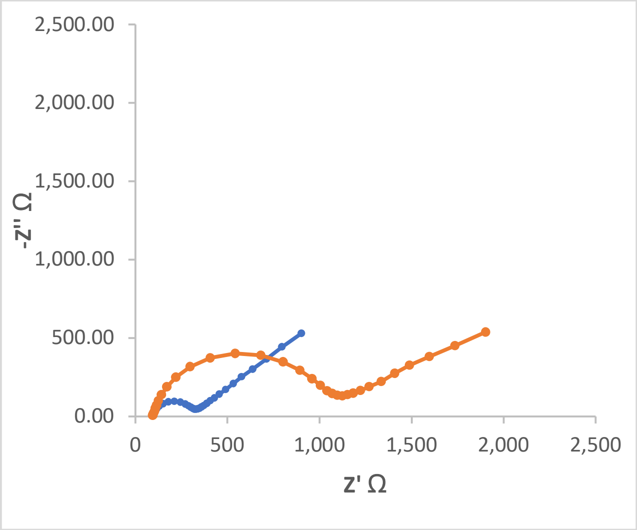

Figure S10: Nyquist plot of GCE-H6 prior (blue) and after (orange) exposure to Cat. domain.

Nyquist Plot of AuE-H6 with Cat domain and CBM domain:

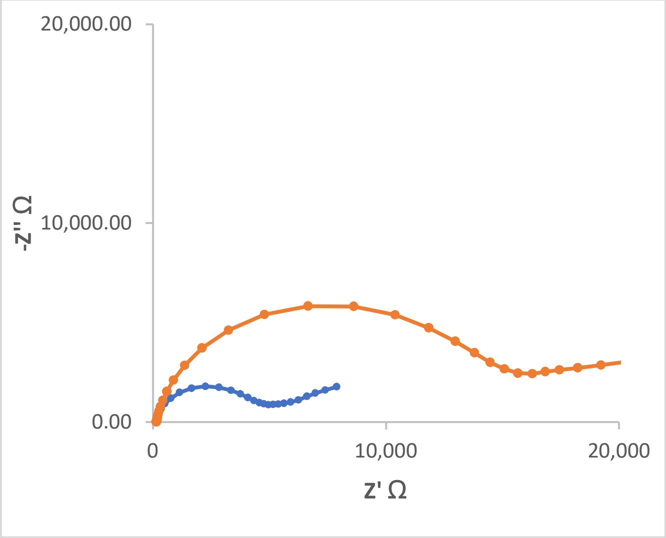

Figure S11: Nyquist plot of AuE-H6 prior (blue) and after (orange) exposure to CBM domain.

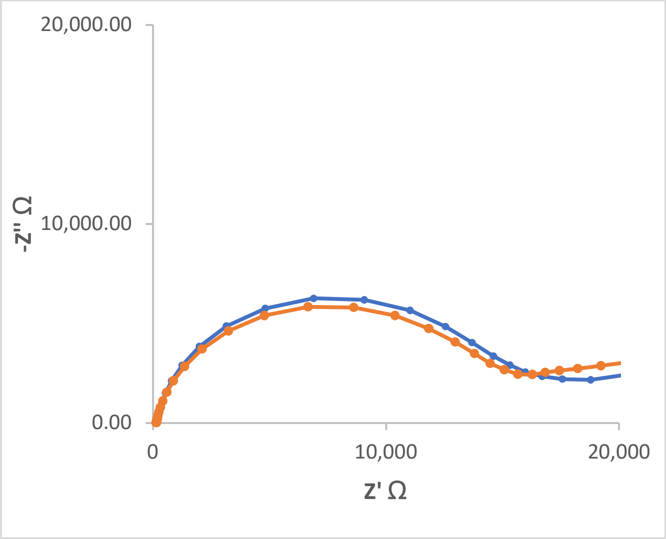

Figure S12: Nyquist plot of AuE-H6 prior (blue) and after (orange) exposure to Cat. domain.

Nyquist Plot of AuE-H6 with Diluted Cat domain:

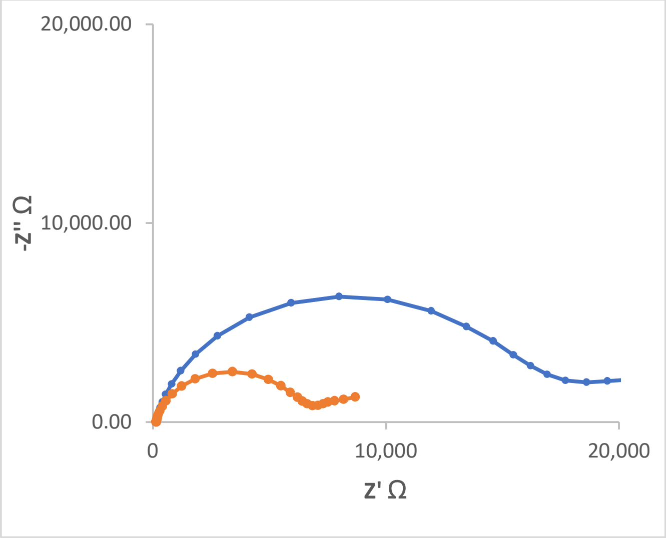

Figure S13: Nyquist plot of AuE-H6 prior (blue) and after (orange) exposure to 100 ng/mL Cat. domain.

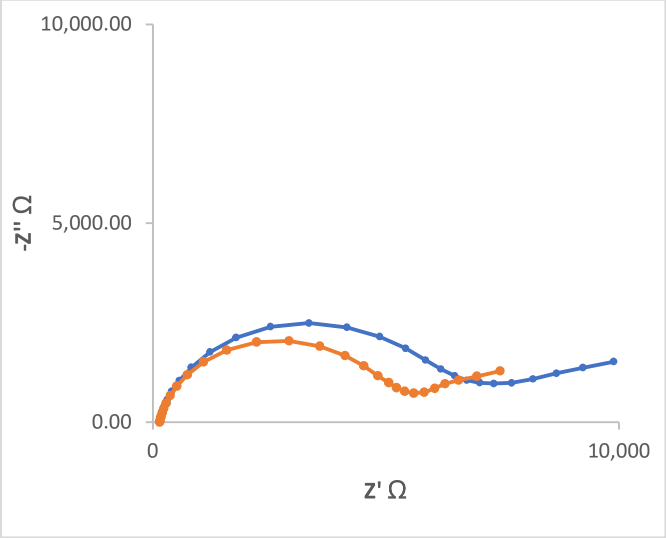

Figure S14: Nyquist plot of AuE-H6 prior (blue) and after (orange) exposure to 1 ng/mL Cat. domain.

Nyquist Plot of AuE-H3 with Cat domain:

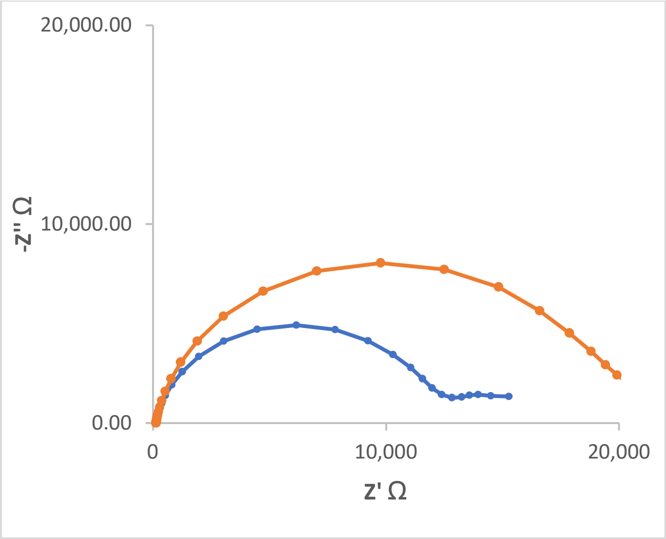

Figure S15: Nyquist plot of AuE-H3 prior (blue) and after (orange) exposure to 10000 ng/mL Cat. domain

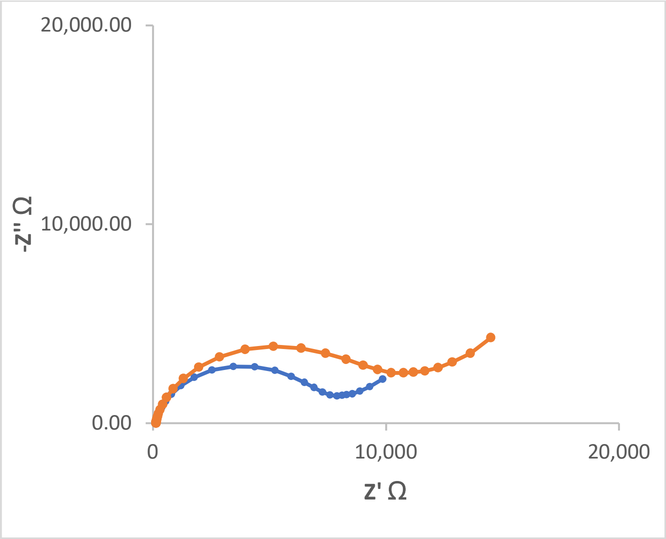

Figure S16: Nyquist plot of AuE-H3 prior (blue) and after (orange) exposure to 100 ng/mL Cat. domain

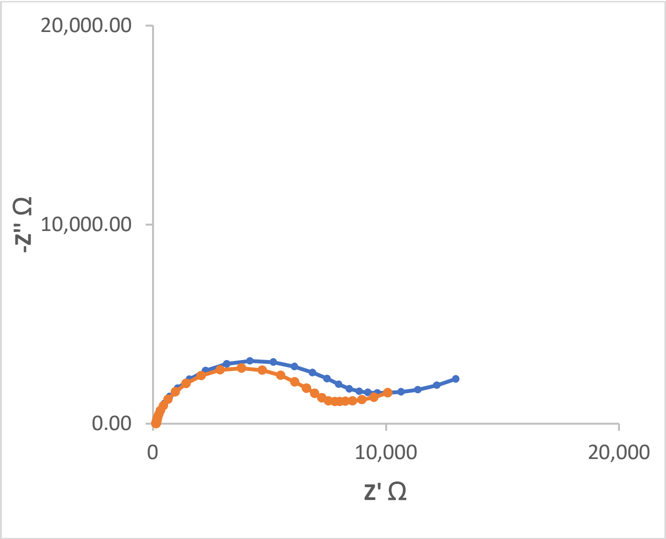

Figure S17: Nyquist plot of AuE-H3 prior (blue) and after (orange) exposure to 1 ng/mL Cat. domain
